# New Alginate/PNIPAAm Matrices for Drug Delivery

**DOI:** 10.3390/polym11020366

**Published:** 2019-02-20

**Authors:** Catalina N. Cheaburu-Yilmaz, Catalina Elena Lupuşoru, Cornelia Vasile

**Affiliations:** 1 Department of Physical Chemistry of Polymers, “Petru Poni” Institute of Macromolecular Chemistry of the Romanian Academy, 700487 Iași, Romania; 2 Department of Pharmacology, Faculty of Medicine, “Grigore T. Popa” University of Medicine and Pharmacy, 700115 Iaşi, Romania; celupusoru@yahoo.com

**Keywords:** alginate, poly(*N*-isopropyl acryl amide), interpolymeric complex, graft copolymer, theophylline, drug delivery, biocompatibility

## Abstract

This paper deals with a comparative study on the interpolymeric complexes of alginate poly(*N*-isopropyl acryl amide (PNIPAAm) and corresponding graft copolymers with various compositions in respect to their toxicity, biocompatibility and in vitro and in vivo release of theophylline (THP). Loading of the various matrices with theophylline and characterization of loaded matrices was studied by near infrared spectroscopy–chemical imaging (NIR–CI) analysis, scanning electron microscopy (SEM) and thermogravimetric analysis (TGA). It was appreciated that THP loading is higher than 40% and the drug is relatively homogeneous distributed within all matrices because of some specific interactions between components of the system. All samples have been found to be non-toxic and biocompatible. It was established that graft copolymers having a good stability show a better drug carrier ability, a higher THP loading, a prolonged release (longer release duration for graft copolymers of 235.4–302.3 min than that for IPC 72/28 of 77.6 min, which means approximately four times slower release from the graft copolymer-based matrices than from the interpolymeric complex) and a good bioavailability. The highest values for THP loading (45%), prolonged release (302.3 min) and bioavailability (175%) were obtained for graft copolymer AgA-*g*-PNIPAAm 68. The drug release mechanism varies with composition and architecture of the matrix.

## 1. Introduction

In the past decade the development of industry and industrialized activities have generated not only concerns about environmental pollution but also about people’s health [1]. This was reflected in the increase of the cases of people from all age categories showing health problems due to environmental contamination, especially oral mucosal and respiratory diseases. Generally, for alleviation and treatment of these diseases, oral, parenteral or pulmonary delivery methods are the most frequently applied. Generally, when a route administration is selected, for a very efficient therapeutic effect a lower administered dose and reduced side effects should be accomplished [1,2,3].

A great challenge among the currently used drugs for these purposes is the burst release of the therapeutical agent from the formulations which could lead to side effects concerning an inefficient therapy with further effects at the tissue level and in blood concentrations. To obtain a controlled, prolonged and targeting drug release, various kinds of matrices have been proposed as: electrospun polymer matrices, electrospun polymer patches, and nanofibres [4,5], hydro (gel) microspheres and nanogels [6,7,8], drug-conjugated thermogels [9], etc.

The development of delivery systems has accelerated in the past decade, as new and modern synthesis and characterization methods were implemented. Polymers were reported as being successful carriers for drug delivery, among them natural polymers e.g., albumin, carrageenan, chitosan (CS), gelatin, hyaluronic acid, alginate, synthetic polymers e.g., poly(lactic acid), poly(vinyl alcohol), acrylic acid derivatives, poly(lactic-*co*-glycolide acid) (PLGA) copolymers etc., are widely used [10].

Particular attention has been given to alginate-based formulations. Tonnesen and Karlsen [11] reported alginate as being one of the most versatile biopolymers for drug delivery applications and not only that. If the conventional formulations with alginate had properties like thickening, gel forming ability or as a stabilizer of the excipients, demand for prolonged action of the therapeutic effect led to development of alginate-based matrices, producing tailor-made products. Its ability to form gels, both pH-dependent and ionotropic gels, combined with the properties of other moieties e.g., stimuli responsiveness could provide new hybrid systems which may comply with pharmaceutical needs. Sosnik reviewed the applications of alginic acid and alginates reporting as mucoadhesive biomaterials, their good cytocompatibility and biocompatibility, biodegradation, sol-gel transition properties, and chemical versatility which favor chemical modifications in order to tailor the final properties to make them suitable for pharmaceutical applications [12]. Additionally, it was mentioned in some detailed reports on regulatory aspects that the U.S. Food and Drug Administration (US-FDA) recognized alginate as a “Generally Referred As Safe” (GRAS) material [12,13].

Various architectures of alginate-based systems were studied. The chemical versatility of alginate structure to be further modified to obtain tailored properties was the subject of many reports [14].

Theophylline (THP) is a dimethylated xanthine drug, which is a potent bronchodilator with anti-inflammatory, bronchoprotective and immunomodulatory effects. It is widely used in the treatment of acute asthma and for chronic obstructive pulmonary disease. Theophylline is used to prevent and treat wheezing, shortness of breath, and chest tightness caused by asthma, chronic bronchitis, emphysema, and other lung diseases. It relaxes and opens air passages in the lungs, making it easier to breathe. It is metabolized extensively by the liver mainly by the microsomal system, while the metabolites are eliminated with the urine [15]. When it is orally administrated, from some matrices and for certain patients some side effects such as nausea/vomiting, stomach/abdominal pain, headache, trouble sleeping, diarrhea, irritability, restlessness, nervousness, shaking, or increased urination may occur. The proper selection of the drug carrier is an important point.

Various architectures of alginate modified with poly(*N*-isopropyl acrylamide) (PNIPAAm) were previously prepared e.g., interpenetrated networks [16,17], interpolymeric complexes [18,19] and graft copolymers [20,21,22] to obtain temperature-responsive materials. Their dual responsiveness of reacting to the variation of external stimuli i.e., by shifting of lower critical solution temperature (LCST) to one closed to body temperature and pH-sensitivity was obtained by combining the properties of alginate with the thermoresponsive behavior of PNIPAAm. Interpolymeric complexes (IPC) and graft copolymers of PNIPAAm with alginate (AgA) also behave as “smart” dual-responsive materials showing a dual transition of temperature/pH values in the vicinity of the physiological ones. That is why they have been proposed for applications in drug delivery with promising results [20,23]. The results obtained in our previous papers [18,19,20,21,22,23,24] on the physical-chemical characterization of the alginic acid and poly(*N*-isopropyl acryl amide) IPC and graft copolymers demonstrated their ability to act as drug carriers. The synthesis method of the graft copolymers was based on the coupling reaction of amino-terminated poly(*N*-isopropyl acryl amide) (PNIPAAm-NH_2_) in the presence of 1-3-(3-dimethylaminopropyl)–3-ethyl-carbodiimide hydrochloride as a condensing agent and 1-hydroxibenzotriazole hydrate (HOBt) as a coupling agent [25]. This kind of synthesis method allows for controlling the structure, the grafted PNIPAAm chain length, and the grafting degree.

In the present study the comparative in vitro/in vivo release behavior of THP from matrices constituted from an alginate (AgA)/PNIPAAm interpolymeric complex (IPC 72/28) and three AgA-*g*-PNIPAAm graft copolymers (C25, C43 and C68) was investigated. The active substance used was THP, well-known for its therapeutic effect in the case respiratory disease treatment such as bronchodilator activity; improvement in forced expiratory volumes; reduction in the frequency of asthmatic attacks, etc. In vivo and in vitro release profiles and kinetics and also the results on toxicity and biocompatibility were presented.

## 2. Experimental

### 2.1. Materials

A commercial alginic acid purchased from Fluka (Honeywell International Inc., by VWR International GmbH, Wien, Austria), was used. It has an average molecular weight of 48,000–186,000 Da; the reduced viscosity in water at 25 °C for a 0.2 wt % aqueous solution was determined as being η_red_ ~2.41 mL·g^−1^ with a drying loss ≤ 10 wt % and ash content of ~3 wt %. The amino-functionalized PNIPAAm was synthetized in laboratory [26,27]. The NIPAAm monomer was dissolved in water and its polymerization took place in presence of potassium persulfate and 2-aminoethanethiol hydrochloride (Sigma-Aldrich Chemie GmbH, Export Department, Taufkirchen, Germany) as an accelerator at 29 °C for 3 h. It has an number average molecular weigth of *M*_n_ 15 kDa. Using these components two sets of matrices were selected for study:

1. An interpolymeric complex (IPC) of AgA/PNIPAAm as previously described by solutions mixing using “iso pH” method [18,19]. The diluted aqueous both polymer solutions of equal concentration (0.5 wt %) and at the same pH (e.g., of 5.5) were mixed in various ratios (15/85; 30/70, 41/59 and 72/28 wt % AgA/wt % PNIPAAm). These ratios were previously established as the most stable associations by hydrogen bonding. The freeze-dried IPCs of various compositions were separated from solutions. The optimal composition AgA/PNIPAam 72/28 (*w*/*w* %) (IPC 72/28) was selected for this study.
2. Graft AgA-*g*-PNIPAAm copolymers were synthesized and characterized as reported in previous studies [21,22,24] by using a method similar with that applied for other types of copolymers [25,26,27,28]. Grafting of PNIPAM-NH_2_ chains onto sodium alginate, NaAgA, was realized by using 1-3-(3-dimethylaminopropyl)–3-ethyl-carbodiimide hydrochloride (EDC) (Sigma Aldrich Chemie GmbH, Export Department, Taufkirchen, Germany) 98% as a condensing agent in the presence of 1-hydroxibenzotriazole hydrate (HOBt) (FlukaHoneywell International Inc., by VWR International GmbH, Wien, Austria)) as a coupling agent. The grafting reaction occurred through an amide group formed from the carboxylate groups of sodium alginate (NaAgA) and the amine group of the amine-terminated PNIPAAm. The graft copolymers obtained were purified by several successive precipitations in acetone and finally freeze-dried [21,22,24,28]. The composition of the graft copolymers obtained was assessed by ^1^H nuclear magnetic resonance (NMR) spectroscopy (BRUKER AVANCE DRX 400 MHz apparatus, Billerica, MA, USA), using D_2_O as solvent. The content in PNIPAAm found varied from 30% to 68% and average viscosity molecular weight (*M*_v_) of the PNIPAAm side chains were about 19–35 kDa.

Both the interpolymeric complex AgA/PNIPAAm IPC (IPC 72/28) and the graft copolymers, mainly AgA-*g*-PNIPAAm 43 (C43) and AgA-*g*-PNIPAAm 68 (C68), were selected for biocompatibility study and in vitro/in vivo release behavior of theophylline (THP) (the numbers represent the PNIPAAm content in graft copolymers). Both groups of studied samples are dual-responsive materials showing sudden changes at a certain range of both pH and temperature variation, and therefore their application as matrices for drug delivery is interesting for obtaining prolonged and targeted release.

The final end-products were processed up for the applied tests:

- Suspensions in water and carboxymethyl cellulose (5 wt %) for the in vivo drug release.
- Lyophilized form for the physical chemical characterization.

Anhydrous crystalline theophylline supplied by BASF (Copenhagen, Denmark) was chosen as model drug for the release experiments, purity 97 to 100 wt %.

### 2.2. Preparation Methods

#### Preparation of Theophylline-Loaded Samples

The loading of theophylline within the polymeric matrices (IPC and graft copolymers) was done by mixing the drug with the lyophilized powdered matrices and using as solvent distilled water obtaining a semi-solid formulation. The concentration of theophylline in the polymeric matrix was 5 wt %. The suspensions obtained were freeze-dried using a Labconco FreeZone device (Kansas City, MO, USA), in order to obtain lyophilized sponge-like materials or powders to be further used for the in vitro measurements. 

For the in vivo experiments the drug loading was performed in a similar manner by forming a semi-solid formulation in 1/1 ethanol/water mixture which swells better the polymer network and the solubility of the drug was higher. 

The loading of formed hydrogels was done by adsorption of theophylline as a concentrated solution of 4 wt % (1/1, *v*/*v*, ethyl alcohol/ water). The mixture was mildly stirred for 24 h, to favor the polymeric system to reach the equilibrium swelling degree. The efficiency of the polymeric matrices to load theophylline was tested by means of near infrared (NIR) spectroscopy and high-performance liquid chromatography (HPLC) studies. 

### 2.3. Investigation Methods

The polymeric matrices (IPC and graft copolymers) based on alginate and PNIPAAm and theophylline-loaded matrices were tested for their toxicity and biocompatibility. Additionally, the matrices were characterized on their particle size, thermal behavior, distribution of the drug within the polymeric matrix, morphology, and in vitro and in vivo drug-delivery profiles.

#### 2.3.1. Near Infrared Spectroscopy–Chemical Imaging (NIR–CI)

Near infrared spectroscopy–chemical imaging (NIR–CI) was used to determine the distribution of the theophylline into polymeric matrices and the chemical homogeneity of the unloaded and drug loaded matrices. Samples as powder were measured by means of NIR spectra on a SPECIM’S Ltd. Sisu CHEMA instrument (Spectral Imaging Ltd. Oulu, Finland) controlled with the Evince software package for processing the original image data. The system used was equipped with a Chemical Imaging Workstation for 1000–2500 nm NIR domains. The original image for each sample was taken with a NIR model spectral camera, respectively an imaging spectrograph type ImSpector N17E (Specim, Spectral Imaging Ltd. Oulu, Finland (with 320 and 640 pixel spatial resolution at a rate of 60–350 Hz. 

#### 2.3.2. Scanning Electron Microscopy (SEM)

Microscopic investigations of the unloaded and loaded hydrogels as dried sponges with theophylline were performed. Analyzed samples were firstly frozen by direct immersion in liquid nitrogen and then fractured. After metallization with gold, the sample examination was performed on a Quanta 3D scanning electron microscope (SEM, Thermo Fisher Scientific, Waltham, MA, USA). Magnification is given on the images.

#### 2.3.3. Thermogravimetric Analysis (TGA) 

The thermo-oxidative behavior of the IPC and grafted copolymers of AgA/PNIPAAm loaded with theophylline was evaluated by thermogravimetric analysis (TGA) using a Q 500 (TA Instruments, New Castel, DE, USA) thermal analyzer. For each recording, 5 mg of sample was placed in Al_2_O_3_ crucibles. The crucibles were placed in a small electrically heated oven with a thermocouple to accurately measure the temperature ensuring high precision in weight, temperature, and temperature change. Temperature calibration was done with standard indium, zinc, tin, bismuth, and aluminum of 99.99% purity. The thermoravimetry was performed in an air/autogenerated atmosphere, at a heating rate of 20 °C/min, in the 15–700 °C temperature range. The characteristic temperatures determination from derivative TGA (DTGA) curves has a reproducibility of ±2 °C. 

#### 2.3.4. Ethics Statement for Experiments with Animals

The experiments on animals were performed according to the institutional guidelines for care and use of laboratory animals and the experimental research protocol was approved by the Animal Research Ethics Committee of “Grigore T. Popa” University of Medicine and Pharmacy of Iasi, Romania (official paper no. 15559/21.09.2010) in rigorous accordance with international ethical regulations on laboratory animal work.

The laboratory animals were anesthetized by exposure to diethyl ether vapors via the respiratory route by exposing them to ether overdose for approximately 2 min in transparent acrylic closed containers. The experiments were performed in compliance with safety regulations [29,30]. Groups of six animals were used for each kind of experiment.

#### 2.3.5. Toxicity and Biocompatibility Studies

Biocompatibility of these systems was tested within the Laboratories of “Grigore T. Popa” Medicine and Pharmacy University, Iasi, Romania. The toxicity and specific biocompatibility tests were performed on white Swiss strain male mice (25–30 g) by determining the rate of living of white Swiss mice after intra-peritoneal injection of the suspensions of polymeric matrices loaded with THP in carboxymethyl cellulose 0.5 wt %. Specific parameters like haemogram, the phagocytic capacity of neutrophils (NBT test), the opsonic capacity of serum, the phagocytic and bactericidal capacities of peritoneal macrophages and the hepatotoxic effects by determining the enzymatic levels of aspartate aminotransferase (AST), serum alanine aminotransferase (ALT) and lactate dehydrogenase (LD) were determined. Details on the methodology used for performing these tests were reported in previous similar studies applied on different matrices [23,31,32].

#### 2.3.6. Statistical Analysis

The triplicate tests were performed and the obtained results are expressed as means ± SD (standard deviation) and significance was analyzed using the T-student test in Microsoft Excel for Windows.

The Stat View statistical software package (Apple Macintosh, Brain Power Inc., Cary, CA, USA) was used for data analysis. Analysis of variance (ANOVA) and Fisher’s post hoc test consisting of 3 (groups), 3 (time sample points) repeated measures of experimental results were analyzed. The criterion for significance was *p* < 0.05.

#### 2.3.7. In Vitro Theophylline Release

The in vitro release studies for theophylline have been performed by a standard dissolution test carried out in conditions which mimic the gastrointestinal environment, using acidic buffer solution of pH 2.2 as dissolution medium. During the experiment, the temperature was maintained at 37 ± 0.5 °C. Aliquots of the medium of 1 mL were withdrawn periodically at predetermined time intervals and analyzed using a HP 8450A ultraviolet (UV)–Visible spectrophotometer (Hewlett Packard, Palo Alto, CA, USA). Recordings were made at λ = 271 nm the wavelength characteristic to theophylline. In order to maintain constant the measured drug concentration, the sample was carefully reintroduced in the circuit after analysis.

The concentrations of the drugs were calculated based on calibration curves predetermined for the drug at specific maximum absorption wavelength mentioned above. 

The drug release kinetics, Korsmeyer–Peppas semi-empirical Equation (1) was applied for the initial release stages (~60% fractional release) [33]:

*M*_t_/*M*_∞_ = *k*_r_*t*^*n*_r_^ (1)

where *M*_t_/*M*_∞_ is the fractional drug released, *M*_t_ and *M*_∞_ are the cumulative drug released amounts at time *t* and at equilibrium, respectively (or experimental maximum released amount taken at the plateau of the release curves), *k*_r_ is rate constant dependent on the characteristics of the drug loaded system, and *n*_r_ is the diffusional exponent which defines the type of the release mechanism. For example, a value of *n*_r_ ~0.5 is characteristic of the Fickian diffusion mechanism of the drug from the cryogel; the values in interval 0.5 < *n*_r_ < 1 are specific to an anomalous or non-Fickian behavior. A case II of the transport mechanism occurs when *n*_r_ = 1, which means zero-order kinetics, while a special case II of the transport mechanism is indicated by values *n*_r_ > 1 [34] The THP release profiles are plotted as the cumulative percentage of drug released versus time.

#### 2.3.8. In Vivo Theophylline Release

The in vivo release of raw theophylline and theophylline loaded within the two types of matrices with alginate (IPC 72/28 and C68) were studied. Selection of loaded matrices for in vivo testing was firstly done based on the outcomes obtained from previous tests, more particularly in vitro tests and loading capacity determined by NIR analysis. In vitro release profiles of the three graft copolymers of alginate with PNIPAAm were almost similar. From the three graft copolymers, C25, C43 and C68, the C68 one with higher amount of PNIPAAm showed better capacity to load THP, and therefore it was selected to be in vivo tested.

In vivo release behavior of THP was investigated on adult Wistar rats and drug serum concentration was determined by HPLC assay. An HPLC (Shimadzu Model-CTO-20A HPLC system, Osaka, Japan) was used. The separation was performed on a 5 μm ZORBAX SB-C18 column (150 mm × 4.6 mm i.d.) in the following conditions: mobile phase composed of acetonitrile10 mM aqueous sodium acetate (7:93 *v*/*v*) under a flow rate of 1 mL/min at room temperature, injection volume of 100 μL; THP was detected by UV detector at 270 nm. A calibration curve with standard solutions of known THP concentrations (1–20 μg/mL) in ethyl alcohol/water (1:1 *v*/*v*) was drawn. The software LC Solution Version 1.22 SP1 was used for integration and automatic determination of drug concentration in blood samples. Other details on experiments and on the pharmacokinetics and in vivo release studies were performed similarly as previously reported for other polymeric matrices [23,31,32]. 

Oral delivery was chosen as it is a common method of drug administration. The animals were maintained in identical laboratory conditions as before, with free access to food and water. The raw theophylline and theophylline-loaded matrices were delivered by gastro-gavage as suspensions, in a dose of 15 mg/kg body weight for each rat [35]. The in vivo release protocol and methodology were detailed as well in our previous studies [23,31,32]. The pharmacokinetic (PK) parameters such as maximum plasma concentration (*C*_max_), time of maximum concentration (*t*_max_) and plasma elimination half-life (*t*_1/2_) were obtained directly from the plasma concentration–time plots. The area under the plasma concentration-time curve up to 72 h (AUC_0–72_) was calculated using the linear trapezoidal rule. The relative bioavailability (test/reference ratios) of the chitosan based hydrogel formulations, compared to raw theophylline was calculated as the ratio (AUC_sample_/AUC_theophylline_) × 100. Each experiment was repeated four times and the results were represented by means of standard deviation.

## 3. Results and Discussions

**I. Evaluation of Physical Chemical Characteristics**

### 3.1. Naer Infrared (NIR) Results

Theophylline distribution into AgA/PNIPAAm systems (IPC and graft copolymers) was evaluated by the NIR–CI technique. The chemical imaging provides a simple method for evaluating the spatial drug distribution. The drug-loading degree was evaluated based the near infrared chemical imaging maps.

The non-destructive character and the facile way to prepare the samples promoted this technique as very feasible to investigate materials for pharmaceutical applications. The homogeneity and prediction of the two constituents of the system (i.e., drug and the polymeric matrix) were assessed by applying two mathematical models, particularly, PLS-DA (partial least squares-discriminate analysis) and PCA (principal component analysis). The PLS-DA model based on the multivariate inverse least squares discrimination method is used to classify the components by Evince software. To each constituent a color was assigned, and applying the aforementioned model the assessment of the degree of homogeneity of the components was undertaken. A value between 0 and 1 of the same original component was assigned and facilitated to obtain quantitative information. For the first component, value 1 represents available percentage of 100% and the value tends to zero when there is an unknown structure. It is appreciated the correct information available upon the cube of information can be extracted. The final images have for every pixel a complete spectrum that includes contributions from all the chemical components present in system. 

**Figure 1:**
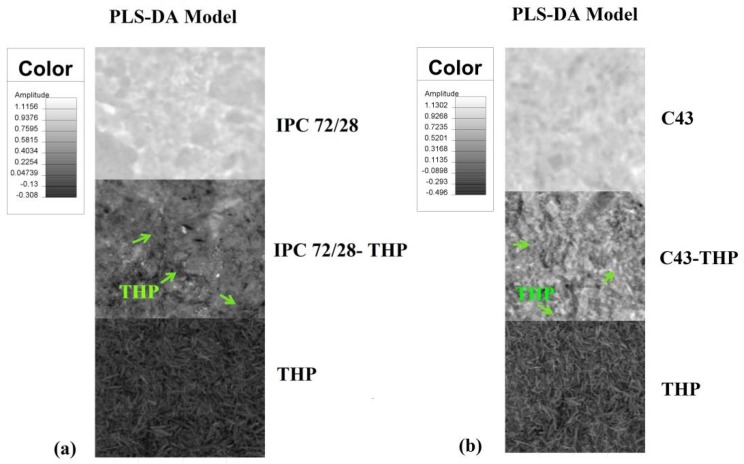

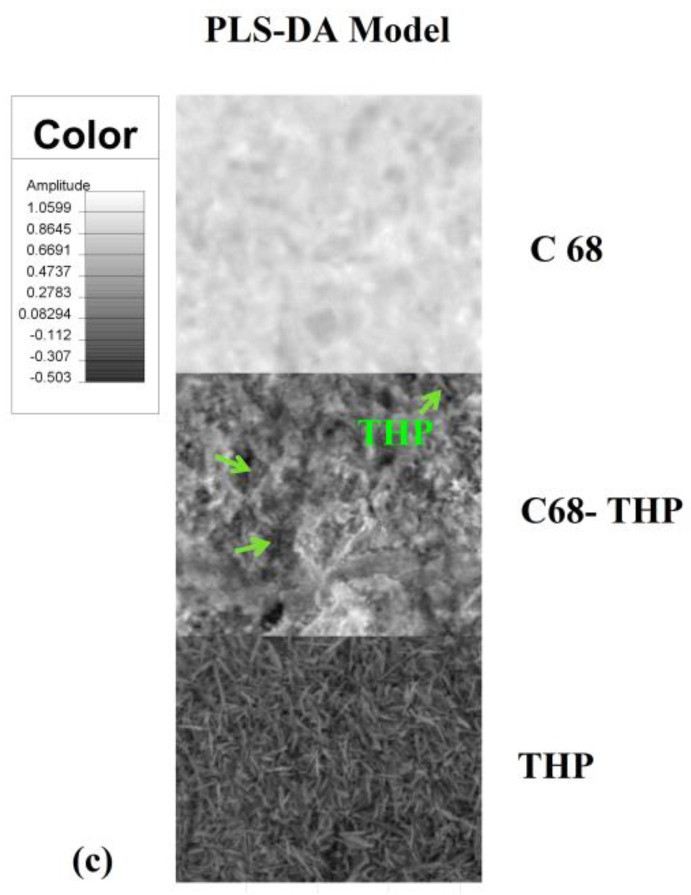
Partial least squares-discriminate analysis (PLS-DA) model images obtained for (**a**) interpolymeric complexes (IPC) 72/28 (**b**) C43 and (**c**) C68 copolymers.

The images in Figure 1 correspond to the PLS-DA model for IPC 78/28 and two copolymers AgA-*g*-PNIPAAm C43 and C68. A visibly uniform distribution, corresponding to a high homogeneity degree of the drug in the matrices was observed. Based on the PLS-DA prediction, a drug loading up to 60% into the IPC and 45% into AgA-*g*-PNIPAAm copolymers was found from the theophylline-loaded amount—Table 1.

**Figure 2:**
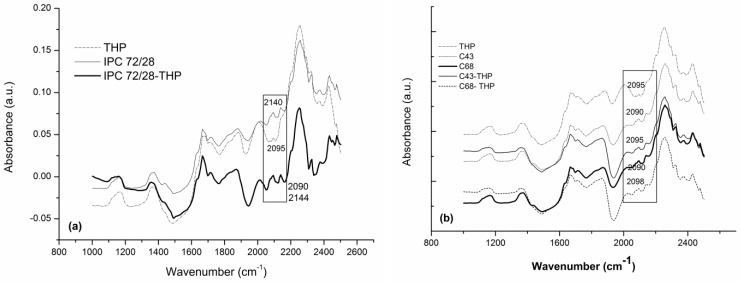
NIR spectra of theophylline, for IPC 72/28 (**a**) and AgA-*g*-PNIPAAm, C43 and C68 (**b**).

According to the data obtained, a quantity of 1.53–1.8% unknown compound is detected which indicate some interactions between components of the systems. In Figure 2a,b are shown the NIR spectra of theophylline, for IPC 72/28 (Figure 2a) and AgA-*g*-PNIPAAm, C43 and C68 (Figure 2b) unloaded and loaded with theophylline in the full range of the near infrared region.

The most visible differences between the loaded and unloaded matrices were observed in the 2095 and 2140 cm^−1^ spectral region which were assigned to the O–H, N–H combinations’ stretching vibrations, confirming the presence of the drug within the matrices and its interaction with the polymeric moieties via H-bonds. Additional information regarding the assignments of the bands is included in Table 2. The most characteristic bands of THP are distinctly identified in loaded matrices or they are shifted to the lower wavenumber indicated as already mentioned, the interactions between components in all three cases—Table 2. In particular, theophylline showed more visible specific bands at about 2475 nm corresponding to the C–N–C bond.

The bands corresponding to the functional groups of IPC and the copolymers are mostly overlapped with those ones of theophylline.

### 3.2. Scanning Electron Microscope (SEM) Images

**Figure 3:**
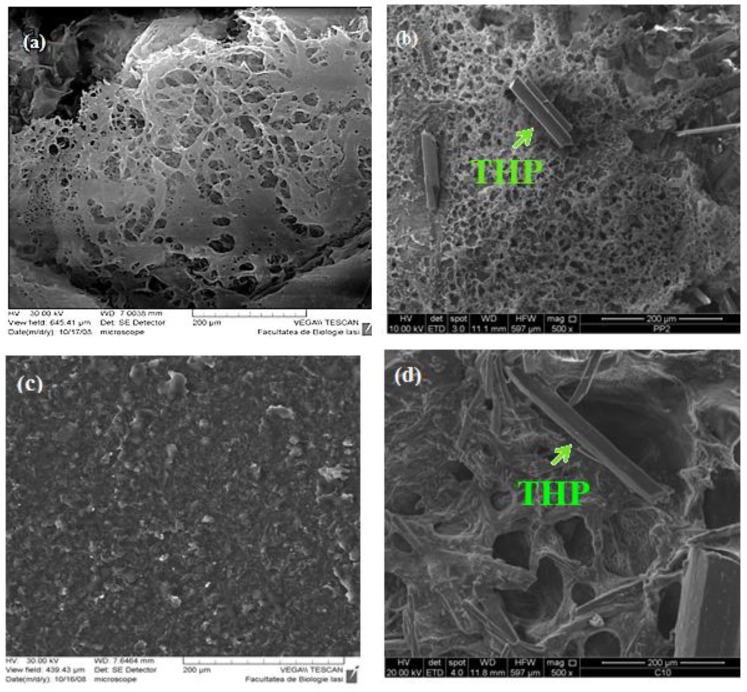
Scanning electron microscope (SEM) images of IPC 72/28 unloaded (**a**) and loaded with theophylline (**b**) and grafted copolymer AgA-*g*-PNIPAAm unloaded (**c**) and loaded with theophylline (**d**).

Polymeric matrices, both IPC and graft copolymers had a porous structure due to the lyophilization. The SEM images (Figure 3a–d) confirmed that theophylline as a crystalline drug was dispersed within the polymeric matrices as stick-shaped like microparticles. The various dimensions of the theophylline particles dispersed between the polymeric matrices were determined and they were in the range of 10 to 50 μm.

SEM images confirmed the NIR-CI results regarding the uniform distribution of theophylline within the polymer matrices.

### 3.3. TGA Results

**Figure 4:**
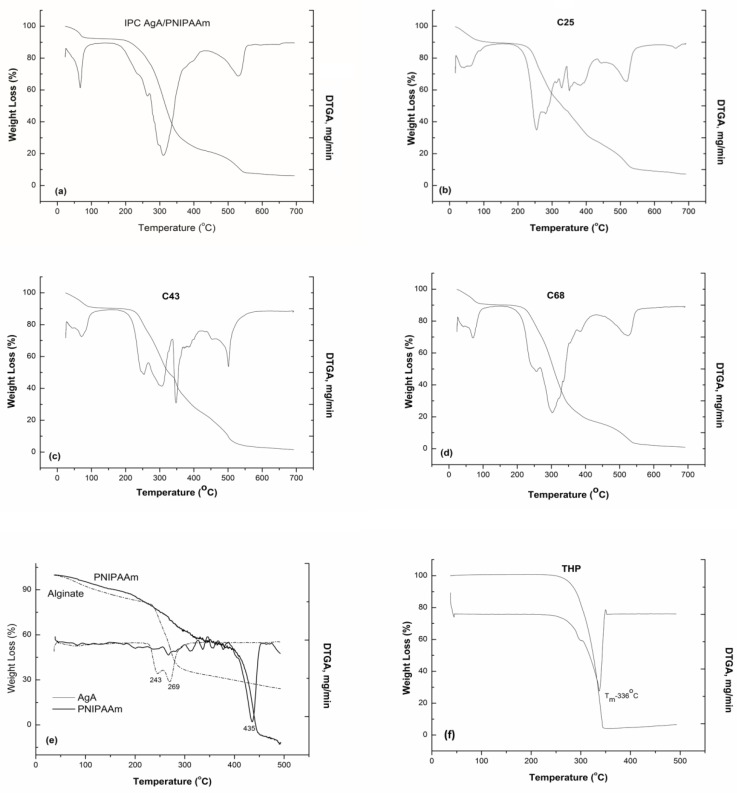
TG and derivative (DTGA) curves of IPC 72/28 (**a**), grafted copolymers (C27—**b**, C43—**c**, C68—**d**), polymeric components, alginate and PNIPAAm (**e**), and pure theophylline (**f**).

The thermal characteristics were studied by means of thermogravimetry. The thermogravimetric analysis was undertaken in order to evidence presence of drug in matrices and also the interactions taking place between the polymeric matrices and loaded drug. This information is obtained by studying of any physical and chemical processes occurring by the application of heat or during storage. Figure 4a–f shows the TGA and their derivative (DTGA) curves obtained for various alginate/PNIPAAm matrices unloaded and loaded with theophylline. Additionally, the thermal behavior on the degradation of pure alginate and THP were studied for a better understanding of the degradation of the more complex systems (IPC and graft copolymers).

By comparative analysis of the TG/DTGA curves of theophylline loaded samples and pure polymer and drug, it was observed that weight loss of the loaded polymeric matrices occurred up to 700 °C in six overlapping steps which corresponded to the individually degradation of the components of the systems. Table 3 summarizes the main thermal characteristics determined from these TG/DTGA curves.

The first step, occurring within the range 37–60 °C, could be assigned to the moisture loss which counts for about 8 to 11 wt %. The dehydration process is followed by several successive degradation steps, cumulating a mass loss of 65% to 80% corresponding to the main decomposition processes of alginate between 250 to 270 °C, drug entrapped which decomposes at about 290–340 °C, PNIPAAm decomposition at about 400 °C. As observed, the DTGA curves of the loaded samples showed a complex decomposition process between 250 and 540 °C, due to the drug–polymeric matrix interactions and polymer–polymer interactions. With the addition of THP within the polymeric matrices (IPC and graft copolymers), the thermal stability was increased in respect of that of alginate. Similar results for degradation of semi-IPN alginate/PNIPAAm were obtained, the outcome of improved thermal behavior of the drug loaded polymeric matrices being confirmed [23]. 

Because the peaks in DTG overlap, the weight loss corresponding to each step is not possible to evaluate correctly.

### 3.4. Toxicity and Biocompatibility Evaluation

#### 3.4.1. Toxicity Tests

The toxicity test supposed the intraperitoneal (i.p.) administration of a single dose of 2000 mg polymer suspension per kg body at a mouse, of each type of formulation [35]. Because the mice survived up to 2 weeks after administration, four other mice were injected for each composition, and their survival rate was assessed. It was found that all mice survived 14 days after i.p. administration of polymeric suspensions. No behavioral or physical changes such as abdominal swelling were observed in treated mice following injection or on subsequent days. Throughout the study period, animals showed no signs of peritonitis, lethargy, muscle loss, dehydration or anorexia, symptoms which are associated with animal toxicity [36]. The acute toxicity of a 5000 mg/kg [35] body dose was tested according to Organization for Economic Cooperation and Development (OECD) guidelines [37]. Due to technical problems occurred at administration of the solutions of such a high concentration, one single dose of 3200 mg kg body was tested. It was found that after a single dose of 3200 mg /kg body of polymeric suspension, intra-peritoneal administered to mice, for IPC 72/28 and AgA-*g*-PNIPAAm C43 and C68; they survived 14 days after administration.

The LD50 for IPC 72/28-THP and AgA-*g*-PNIPAAm C43 and C68-THP, after intraperitoneal administration of suspensions, was found to be higher than 3200 mg/kg. The obtained LD50 values demonstrated that the compositions have low toxicity.

#### 3.4.2. Biocompatibility Studies

The in vivo biocompatibility of IPC 72/28 and C43 and C68 graft copolymers was examined during 14 consecutive days after i.p. injection of polymeric suspensions at mice. The effects on hematological and immune system parameters were followed comparatively with a control group of mice, which received simple physiological serum. The determined values of the hematological and immune systems parameters are listed within Table 4. Clinical chemistry and hematological data are of great importance to determine the effects induced on the body by the tested hydrogel matrices. The values of hematological parameters such as white blood cells (WBC), red blood cells (RBC), platelets (PLT), hemoglobin level (HGB) concentration, hematocrit level showed no significant variations between mice groups treated with IPC 72/28-THP and C-THP hydrogels compared with control mice group, being in the range of normal limits reported for healthy mice [38]. Additionally, the values obtained for these systems were comparable with similar matrices from alginate and PNIPAAm as semi-interpenetrated networks [23]. Statistical analysis revealed no significant influence of the studied compounds on the neutrophils phagocytic capacity, on the phagocytosis activity of immune cells, serum opsonic capacity, phagocytosis and bactericidal capacities of peritoneal macrophages, splenic lymphocytes with rosetting capacity of tested mice compared to the control group after 14 days of testing.

Based on the toxicity and biocompatibility results it can be considered that the IPC 72/28 and C68 graft copolymer based formulations are nontoxic and did not produce any significant changes in the hematology of tested rats and had no hepatic-toxicity effect. All presented results indicated a good biocompatibility of IPC 72/28 and C68 graft copolymer with living tissues, and so they could be considered as potential carriers for controlled drug delivery and other biomedical applications. 

**II. Theophylline Release Studies**

### 3.5. Drug Release Results

#### 3.5.1. In Vitro Release of Theophylline

**Figure 5:**
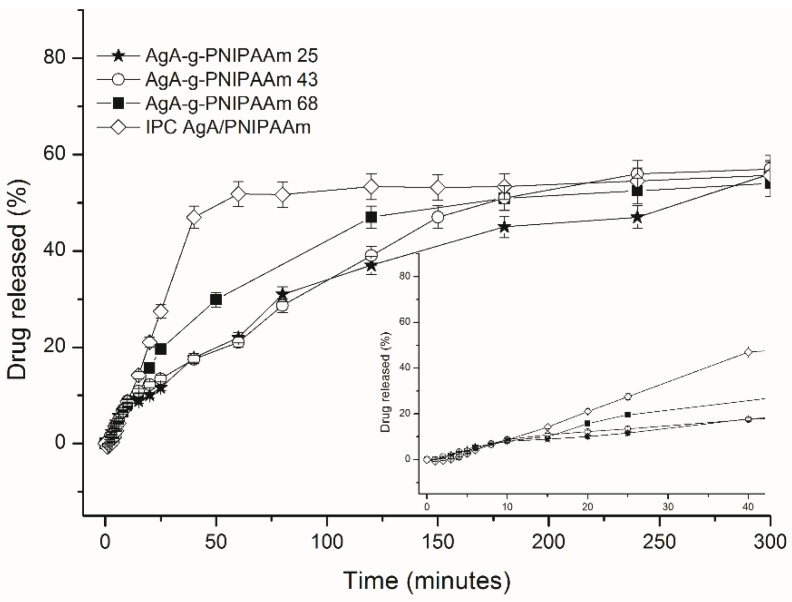
In vitro release profiles of theophylline from matrices constituted from IPC 72/28 and C25, C43 and C68 graft copolymers.

The release profiles of theophylline, as the dependence of the theophylline released as percent vs time, from the matrices of alginate and PNIPAAm with different architectures (IPC and graft copolymers) are shown in Figure 5.

The THP released amount varied between 52.4% and 56.3% with small differences between samples, but they significantly differed in respect of the time to reach equilibrium (*t*_eq_) which was much longer for graft copolymers of 235.4–302.3 min than that for IPC72/28 of 77.6 min. A burst effect was characteristic of THP release from IPC, a large amount of drug being released in the first 25 min. The prolonged released in the case of graft copolymers was observed when amount of PNIPAAm graft copolymer composition was increased. A similar amount of theophylline was released almost four times slower than the graft copolymer-based matrices.

Drug release kinetics was assessed by applying the Korsmeyer–Peppas semi-empirical equation (Equation (1)) for the initial release stages (up to 60% fractional release). Values of release kinetic parameters were summarized within Table 5. 

The diffusional exponent *n_r_* takes value of 1.4 in the case of release of theophylline from IPC matrix and it varied from 0.7 to 1.8 in the case of graft copolymers and it increases with PNIPAAm content of copolymer. Gradual increase of diffusional exponent with the increase of the PNIPAAm content could indicate a different release mechanism. A special case II of transport mechanism is indicated by values *n*_r_ > 1 which is characteristic of THP release from IPC72/28 and C68. Particularly, THP release from C25 occurs by an anomalous diffusion transport *n*_r_ = 0.7 while a zero order kinetics (*n* = 1) is specific for the THP release from C43 copolymer which is known to be the most favorable method for drug delivery. Moreover, the increase of PNIPAAm content could lead to the enhanced sol-gel transition of the copolymer solution at 37 °C due to the temperature responsiveness induced by the PNIPAAm grafted chains. As a consequence, the diffusion of drug is limited by the gelation of the polymer matrix and, thus, has better resistance to erosion and less drug is released. 

It can be concluded that it is possible to control the mechanism of THP release by changes in chemical composition of copolymers. The obtained results are supported also by those obtained in our previous studies where the influence of the physical chemical characteristics of the matrices was studied in much more detail [20,21,22].

As concerns the variation of the rate constant *k*_r_, which also takes characteristic values for each drug-loaded system, it can be observed that it took much lower values for the graft copolymers, which is 10 times slower from C68 than those for other copolymers and IPC 72/28. This could support the prolonged delivery of theophylline from the graft copolymers by comparison with the H-bonded interpolymeric complex based matrix, IPC 72/28. 

#### 3.5.2. In Vivo Theophylline Release

**Figure 6:**
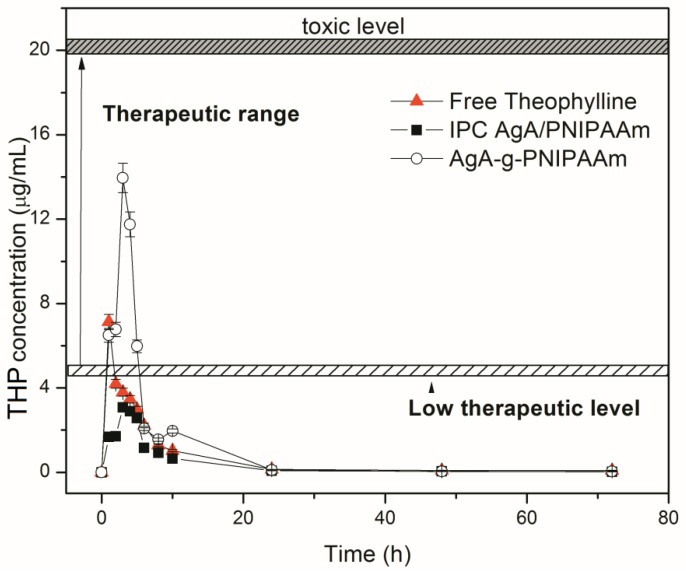
In vivo release profiles of theophylline from IPC 72/28-THP and C68-THP.

The in vivo release of raw theophylline and theophylline loaded within the two types of matrices with alginate (IPC 72/28 and C68) were studied. The mean plasma theophylline concentration versus time curves after a single oral dose are represented in Figure 6 and mean values of pharmacokinetic parameters (*C*_max_, *t*_1/2_, and AUC_0–72_) are summarized in Table 6.

Pure theophylline was detected in plasma within the first hours after its administration in rats. The mean plasma level of raw theophylline was found of *C*_max_ value of 7.1 μg/mL within a time period of *t*_max_ of 1.5 h and the elimination half-life (*t*_1/2_) of about 2.5 h, which indicated a fast absorption of pure theophylline, data that are consistent with previous studies [23]. 

As observed in Figure 6, there was a marked level when theophylline can have a toxic effect, particularly for a concentration of 20 μg/mL [39,40,41,42]. Additionally, it was mentioned that the therapeutic drug level for theophylline is within the range 10–20 μg/mL. A lower amount than 10 μg/mL of delivered theophylline cannot ensure a complete therapeutic effect of the drug and a higher amount in blood plasma may induce a toxic effect. In the case of administration of free drug suspension, it could be observed that the detected concentration in sanguine plasma was up to 10 μg/mL. When theophylline was carried by the polymeric vehicles (IPC and graft copolymer) the changes in the delivery profile were observed. In the case of IPC 72/28-THP, the delivery was prolonged by comparison with free theophylline delivery profile but with a rather low therapeutic level, and thus did not have enough of a therapeutic effect as would be needed. In the case of graft copolymer, theophylline was prolonged released and its concentration was ranged within the therapeutic level reported for theophylline without reaching a toxic level. 

The evaluated pharmacokinetic parameters are given in Table 6. The theophylline release profile showed higher values for the pharmacokinetic parameters in the case of the graft copolymers. This is translated by a retarded release of the drug, as the matrices were more complex and played their role in the drug protection against fast release. Higher bioavailability was also found, especially in the case of graft copolymer-based formulation C68.

## 4. Conclusions

A comparative study on the interpolymeric complex and graft copolymers of AgA and PNIPAAm with various compositions in respect of their toxicity, biocompatibility and in vitro and in vivo release of theophylline was presented. THP-loaded and -unloaded polymeric carriers have been investigated in respect with their structure, morphology, thermal properties, toxicity and biocompatibility. All of them have been found to be non-toxic and biocompatible. It was established that graft copolymers of AgA-*g*-PNIPAAm exhibited better stability due to their structure and also showed a better drug carrier ability, a higher THP-loading ability, a prolonged release, and good bioavailability. The release mechanism varied with the composition and architecture of the matrix. Theophylline was released in a controlled manner and its concentration in sanguine plasma ranged within the therapeutic level reported for theophylline without reaching a toxic level. 

## Figures and Tables

**Table 1:** Evaluation of the theophylline (THP) loading of the IPC and graft copolymers by the near infrared (NIR) method.

Sample	Polymeric Matrix (wt %)	Unknown Compound (wt %)	Drug Loading (%) Raported to Initial THP Amount
IPC 72/28-THP	98.32 ± 4.91	1.68 ± 0.08	42 ± 2.10
C25-THP	98.20 ± 4.90	1.80 ± 0.09	45 ± 2.25
C43-THP	98.47 ± 4.92	1.53 ± 0.07	39 ± 1.95
C68-THP	98.20 ± 4.90	1.80 ± 0.09	45 ± 2.25

**Table 2:** NIR characteristic bands and their assignments for the samples studied.

Theophylline	IPC 72/28		AgA-*g*-PNIPAAm				Assignment
			C43		C68		
	Without THP	With THP	Without THP	With THP	Without THP	With THP	
1170	1163		1160	1152	1160	1152	C–H second overtone
1367	1372	1361	1367	1357			First C–H overtone and combinations
	1433	1437 sh			1441		N–H stretch 1st overtone
	1631 sh	1631 sh	1631 sh	1623s	1635		N–H stretch 1st overtone
1676	1665	1673	1669	1665	1665	1665	C–H stretch 1st overtone
1710	1714	1714s	1718	1707	1722	1711	C–H stretch 1st overtone
1819 1882	1817 sh 1878						C–O and O–H combinations
		1874	1874	1870	1870	1865	
2004	2015	2015	2007s	2007	2011	2015	O–H bend second overtone
2095	2090	2095	2090	2095	2090	2098	O–H and N–H combinations
2137	2140	2144	2136	2140	2140	2144	N–H combinations
2254	2258	2254	2209; 2258	2197; 2258	2197; 2254	2197; 2254	O–H and C–H combinations
2321 2376	2326 2376	2326 2376	2322; 2376	2326; 2376	2322; 2374	2330; 2372	C–H stretch/CH_2_ deformation
2429 2475	2436 2476	2436 2474	2433; 2478	2430	2430; 2478	2429; 2478	C–H and C–C combinations C–N–C stretch overtone

**Table 3:** Maximum temperature from DTGA for THP-loaded IPC, graft copolymers and their components and THP.

Sample	Maximum Temperature (°C)—from DTGA					
	Step 1	Step 2	Step 3	Step 4	Step 5	Step 6
IPC 72/28	68	265	295 sh	312	402 sh	532
C25	37; 60	253	282	311; 327 348; 381	442	517
C43	42; 69	255	306;	350; 385 sh	452	500
C68	42; 69	255	305;	385 sh		524
Alginate		245; 269				
Theophyline			298 sh;	337		
PNIPAAm					436	

sh—shoulder; IPC- interpolymeric complex; C25, C43 and C68—graft AgA-*g*-PNIPAAm copolymers.

**Table 4:** Hematological and immune systems parameters.

Hematological Parameter	Control Mice Group	Tested Mice Groups i.p. Injected with Suspension of:	
		IPC 72/28-THP	C68-THP
White blood cells (×10^9^/L)	5.64 ± 0,13	5.6 ± 0,1	5.65 ± 0.11
Polymorphonuclear cells (PMN) (×10^9^/L)	1.51 ± 0.06	1.51 ± 0.06	1.54 ± 0.04
Lymphocytes (×10^9^/L))	3.68 ± 0.11	3.64 ± 0.09	3.65 ± 0.1
Monocytes (×10^9^/L)	0.35 ± 0.05	0.34 ± 0.05	0.36 ± 0.04
Eosinophils (×10^9^/L)	0.04 ± 0.02	0.05 ± 0.01	0.04 ± 0.02
Basophils (×10^9^/L)	0.05 ± 0.03	0.06 ± 0.01	0.05 ± 0.03
Polymorphonuclear cells (PMN) (%)	26.8 ± 0.97	26.94 ± 1.02	27.25 ± 0.5
Lymphocyte (%)	65.3 ± 1.05	65.34 ± 1.27	64.8 ± 0.52
Monocytes (%)	6.23 ± 0.71	6.14 ± 0.77	6.34 ± 0.56
Eosinophils (%)	0.78 ± 0.36	0.9 ± 0.19	0.74 ± 0.45
Basophils (%)	0.93 ± 0.47	1.08 ± 0.26	0.86 ± 0.54
Red blood cells (×10^9^/L)	9.39 ± 0.06	9.4 ± 0.07	9.41 ± 0.09
Hemoglobin level (g/dL)	11.5 ± 0.05	11.45 ± 0.05	11.48 ± 0.06
Hematocrit level (%)	41.0 ± 0.04	41.08 ± 0.19	41.1 ± 0.2
NBT test (%)	13.8 ± 0.75	13.71 ± 0.76	13.83 ± 0.75
Platelets (×10^9^/L)	253 ± 38.8	252.9 ± 16.2	252.99 ± 8.01
**Immune System Parameters**			
Serum opsonic capacity (*S. aureus* ×1000/mL)	771.7 ± 58.4	774. 3 ± 53.8	773.3 ± 59.55
Phagocytic capacity of peritoneal macrophages *(S. aureus* ×1000/mL)	716.7 ± 51.6	728.5 ± 59.8	735 ± 62.85
Bactericidal capacity of peritoneal macrophages (*S. aureus* ×1000/mL)	696.7 ± 8.2	697.14 ± 9.51	698.33 ± 9.83
Splenic T lymphocytes (%)	12.5 ± 0.55	12.57 ± 0.53	12.67 ± 0.52
TGP (UI/I)	23.17 ± 1.17	24 ± 1.63	23.17 ± 1.17
TGO (UI/I)	73.33 ± 1.75	73.14 ± 1.21	72.33 ± 0.92
LDH (UI/I)	497.5 ± 3.33	498.71 ± 1.8	499.33 ± 3.88

**Table 5:** In vitro drug release characteristics, including kinetic parameters (*n*_r_ and *k*_r_) of THP from IPC and graft copolymers at 37 °C and pH 2.2.

Sample	Maximum Amount Released (%)	*t*_eq._ (min)	*t*_1/2r_ (min)	*n*_r_	*R*_nr_	*k*_r_·10^3^ (min^−1^)	*R*_kr_
IPC 72/28	52.40 ± 2.62	77.6 ± 3.88	25.4 ± 1.27	1.41 ± 0.07	0.98	10.0 ± 0.50	1.00
C25	56.10 ± 2.80	302.2 ± 15.11	73.5 ± 3.67	0.72 ± 0.03	0.96	12.4 ± 0.62	0.97
C43	56.30 ± 2.81	271.4 ± 13.57	82.9 ± 4.14	1.01 ± 0.05	0.96	7.6 ± 0.38	0.97
C68	52.40 ± 2.62	235.4 ± 11.74	41.4 ± 2.07	1.80 ± 0.09	0.93	1.7 ± 0.08	0.97

*t*_eq—_time to reach equilibrium; *t*_1/2r—_time to release a half of drug quantity.

**Table 6:** Pharmacokinetic parameters obtained for raw theophylline and theophylline-loaded matrices.

Parameter	THP	IPC 72/28	C68
*t*_max_ (h)	1.03 ± 0.05	2.90 ± 0.14	3.90 ± 0.19
*t*_½_ (h)	2.50 ± 0.12	7.00 ± 0.35	12.0 ± 0.60
*C*_max_ (μg/mL)	7.10 ± 0.35	3.11 ± 0.15	13.94 ± 0.69
AUC_0–72_ (μg h/mL)	38.52 ± 1.92	22.94 ± 1.14	67.43 ± 3.30
Relative bioavailability (%)	-	59.55 ± 2.97	175.00 ± 8.75

## References

[1] Haque S., Whittaker M.R., McIntosh M.P., Pouton C.W., Kaminskas L.M. (2016). Disposition and safety of inhaled biodegradable nanomedicines: Opportunities and challenges. Nanomed. Nanotechnol. Biol. Med..

[2] Verma M.I.R., Garcia-Contreras L. (2015). Inhalation drug delivery devices: Technology update. Med. Devices Evid. Res..

[3] Carrier J.A., Shaw R.A., Porter R.S., Allison E.J., Kessler E.R., Woody D.G., Harker C.C., Jones J.G. (1985). Comparison of intravenous and oral routes of theophylline loading in acute asthma. Ann. Emerg. Med..

[4] Ding J., Zhang J., Li J., Li D., Xiao C., Xiao H., Yang H., Zhuang X., Chen X. (2019). Electrospun polymer biomaterials. Prog. Polym. Sci..

[5] Li J., Xu W., Li D., Liu T., Zhang Y.S., Ding J., Chen X. (2018). Locally Deployable Nanofiber Patch for Sequential Drug Delivery in Treatment of Primary and Advanced Orthotopic Hepatomas. ACS Nano.

[6] Zhang W., Xu W., Ning C., Li M., Zhao G., Jiang W., Ding J., Chen X. (2018). Long-acting hydrogel/microsphere composite sequentially releases dexmedetomidine and bupivacaine for prolonged synergistic analgesia. Biomaterials.

[7] Zhang W., Ning C., Xu W., Hu H., Li M., Zhao G., Ding J., Chen X. (2018). Precision-guided long-acting analgesia by Gel-immobilized bupivacaine-loaded microsphere. Theranostics.

[8] Li S., Zhang T., Xu W., Ding J., Yin F., Xu J., Sun W., Wang H., Sun M., Cai Z., Hua Y. (2018). Sarcoma-Targeting Peptide-Decorated Polypeptide Nanogel Intracellularly Delivers Shikonin for Upregulated Osteosarcoma Necroptosis and Diminished Pulmonary Metastasis. Theranostics.

[9] Zhang Y., Zhang J., Xu W., Xiao G., Chen X. (2018). Tumor microenvironment-labile polymer–doxorubicin conjugate thermogel combined with docetaxel for in situ synergistic chemotherapy of hepatoma. Acta Biomater..

[10] Liang Z., Ni R., Zhou J., Mao S. (2015). Recent advances in controlled pulmonary drug delivery. Drug Discov. Today.

[11] Tonnesen H.H., Karlsen J. (2002). Alginate in Drug Delivery Systems. Drug Dev. Ind. Pharm..

[12] Sosnik A. (2014). Alginate Particles as Platform for Drug Delivery by the Oral Route: State-of-the-Art. ISRN Pharm..

[13] Chang D., Chang R.-K. (2006). Review of current issues in pharmaceutical excipients. Pharm. Technol..

[14] Sabra W., Deckwer W.-D., Dumitriu S. (2005). Alginate-A polysaccharide of industrial interest and diverse biological functions. Polysaccharides: Structural Diversity and Functional Versatility.

[15] Ogilvie R.I. (1978). Clinical pharmacokinetics of theophylline. Clin. Pharmacokinet..

[16] Dumitriu R.P., Mitchell G.R., Vasile C. (2011). Rheological and thermal behaviour of poly(N-isopropyl acryl amide)/alginate smart polymeric networks. Polym. Int..

[17] Dumitriu R.P., Mitchell G.R., Vasile C. (2011). Multi-responsive hydrogels based on N-isopropylacrylamide and sodium alginate. Polym. Int..

[18] Cheaburu C.N., Vasile C. (2008). Responsive Freeze-Drying Interpolymeric Associations of Alginic Acid and Poly(*N*-isopropyl acrylamide) II. The Dependence of the Transition Temperature on pH and composition. Cell. Chem. Technol..

[19] Duncianu C.N., Vasile C. (2008). Interpolymeric Associations Between Alginic Acid and Poly(*N*-Isopropylacrylamide), Poly(Ethylene Glycol) and Polyacrylamide. Polym. Res. J..

[20] Vasile C., Dumitriu R.P., Cheaburu C.N., Oprea A.M. (2009). Architecture and composition influence on the properties of some smart polymeric materials designed as matrices in drug delivery systems. A comparative study. Appl. Surf. Sci..

[21] Vasile C., Nita L.E. (2011). Novel multi-stimuli responsive sodium alginate-grafted-poly(*N*-isopropyl acryl amide) copolymers: II. Dilute solution properties. Carbohydr. Polym..

[22] Cheaburu C.N., Ciocoiu O.N., Staikos G., Vasile C. (2013). Thermoresponsive sodium alginate-*g*-poly(*N*-isopropyl acryl amide) copolymers. III. Solution properties. J. App. Polym. Sci..

[23] Dumitriu R.P., Oprea A.M., Cheaburu C.N., Nistor M.T., Novac O., Ghiciuc C.M., Profire L., Vasile C. (2014). Biocompatible and Biodegradable Alginate/Poly(*N*-isopropyl acryl amide) Hydrogels for Sustained Theophylline Release. J. Appl. Polym. Sci..

[24] Ciocoiu O.N., Staikos G. (2013). Sodium Algınate-Graft-Poly(*N*-Isopropylacrylamide) Copolymers As Thickening Agents. http://9pesxm.chemeng.ntua.gr/_view_paper/117.

[25] Bokias G., Mylonas Y., Staikos G., Bumbu G.G., Vasile C. (2001). Synthesis and Aqueous Solution Properties of Novel Thermoresponsive Graft Copolymers Based on a Carboxymethylcellulose Backbone. Macromolecules.

[26] Vasile C., Bumbu G.G., Mylonas I., Bokias G., Staikos G. (2004). Thermoresponsive behaviour in aqueous solution of poly(maleic acid-alt-vinyl acetate) grafted with poly(*N*-isopropyl acryl amide). Polym. Int..

[27] Ciocoiu O.N., Vasile C., Staikos G. (2018). Thermoresponsive behavior of sodium alginate grafted with poly(*N*-isopropyl acryl amide) in aqueous media. Carbohydr. Polym..

[28] Cheaburu-Yilmaz C.N., Vasile C., Ciocoiu O.-N., Staikos G., Khutoryanskiy V., Georgiou T. (2018). Sodium alginate grafted with poly(*N*-isopropyl acryl amide). Temperature-Responsive Polymers: Chemistry, Properties and Applications.

[29] AVMA Guidelines for the Euthanasia of Animals. https://www.avma.org/KB/Policies/Documents/euthanasia.pdf.

[30] Zimmermann M. (1983). Ethical guidelines for investigations of experimental pain in conscious animals. Pain.

[31] Popa N., Novac O., Profire L., Lupusoru E., Popa M.I. (2010). Hydrogels based on chitosan–xanthan for controlled release of theophylline. J. Mater. Sci. Mater. Med..

[32] Cheaburu-Yilmaz C.N., Dumitriu R.P., Nistor M.T., Lupusoru C., Popa M.I., Profire L., Silvestre C., Vasile C. (2015). Biocompatible and Biodegradable Chitosan/Clay Nanocomposites as New Carriers for Theophylline Controlled Release. Br. J. Pharm. Res..

[33] Korsmeyer R.W., Lustig S.R., Peppas N.A. (1986). Solute and penetrant diffusion in swellable polymers. I.Mathematical modeling. J. Polym. Sci. Part B Polym. Phys..

[34] Ritger P.L., Peppas N.A. (1987). A simple equation for description of solute release. II Fickian and anomalous release from swellable devices. J. Control. Release.

[35] Ram F.S.F., Jardin J.R., Atallah A., Castro A.A., Mazzini R., Goldstein R. (2005). Efficacy of theophylline in people with stable chronic obstructive pulmonary disease: A systematic review and meta-analysis. Respir. Med..

[36] ASTM International (2017). ASTM F 756-00Standard Practice. Assessment of Hemolytic Properties of Materials.

[37] (2001). OECD/OCDE Guidelines for the Testing of Chemicals, Acute Oral Toxicity—Up-and-Down-Procedure (UDP).

[38] Schneck K., Washington M., Holder D., Lodge K., Motzel S. (2000). Hematologic and serum biochemical reference values in nontransgenic FVB mice. Comp. Med..

[39] Kawai M., Kato M. (2000). Theophylline for the treatment of bronchial asthma: Present status. Methods Find Exp. Clin. Pharm..

[40] Nelson L.S., Ford M.D., Goldman L., Schafer A.I. (2016). Acute poisoning. Goldman-Cecil Medicine.

[41] Pincus M.R., Abraham N.Z., McPherson R.A., Pincus M.R. (2017). Toxicology and Therapeutic Drug Monitoring. Henry’s Clinical Diagnosis and Management by Laboratory Methods.

[42] Diasio R.B., Goldman L., Schafer A.I. (2011). Principles of drug therapy. Goldman-Cecil Medicine.

